# Effects of religious and cultural beliefs on vaccine attitudes in a Hispanic immigrant population in the United States

**DOI:** 10.1371/journal.pgph.0003519

**Published:** 2024-08-06

**Authors:** Ruth J. Larson, Jamie L. Jensen, Scott M. Alvord, Chantel Sloan-Aagard, Ty Skyles, Spencer C. Davis, Acelan M. Obray, Kendall Pogue, Brian D. Poole

**Affiliations:** 1 Department of Public Health, Brigham Young University, Provo, Utah, United States of America; 2 Department of Biology, Brigham Young University, Provo, Utah, United States of America; 3 Department of Spanish and Portuguese, Brigham Young University, Provo, Utah, United States of America; 4 Department of Microbiology and Molecular Biology, Brigham Young University, Provo, Utah, United States of America; Dr. D.Y. Patil Medical College, Hospital and Research Centre, Dr. D.Y. Patil Vidyapeeth, Pune, INDIA

## Abstract

Hispanic Immigrants (HI) have lower vaccination rates than their non-Hispanic white counterparts. Culturally appropriate interventions are more influential in evoking change among viewers; therefore, it is important to understand the cultural factors of specific ethnic groups. In this study, we identify cultural barriers to vaccination of Hispanic Immigrants. An electronic survey was administered among HI parents living in the United States. Using structural equation modeling, we found that high religious practice and positive religious beliefs towards vaccination correspond with positive vaccine attitudes (+0.20 and +0.587, respectively). Trust in institutions is strongly correlated with strong positive vaccine attitudes (+0.734). While trusting one’s folk practitioner more than a medical doctor leads to negative vaccine attitudes (-0.596), the use of home remedies is associated with positive vaccine attitudes (+0.486). The cultural competence of a medical practitioner, especially regarding folk medicine, is essential to lower cultural barriers HI patients face in vaccination.

## Introduction

Vaccines have revolutionized the field of medicine since the late 1700s when they were first popularized. Their significant role in preventing disease and reducing disparities in disease rates around the world is one of public health’s greatest achievements [1]. Vaccines have contributed to the substantial decrease in global infant mortality rate from 65 per 1,000 live births in 1990 to 29 in 2018 [2]. Because of significant scientific progress, children in the US can receive vaccines against 16 diseases [3]. Childhood vaccination has shown to be close to 100% effective in preventing diseases such as diphtheria, measles, mumps, polio, and rubella [4].

Unfortunately, vaccine coverage has decreased significantly over the course of the last decade [5]. Between 2019 and 2021, an increase of 5.9 million children around the world did not receive their routine childhood vaccinations, which marked the largest increase in lack of vaccination since 2009 [6]. This decrease was primarily due to the COVID-19 pandemic [7]. During this same timeframe, overall global vaccine coverage decreased by 5% [8]. Although barriers to vaccine access, such as living in rural communities or not having access to reliable healthcare, can contribute to decreased vaccination rates, the sharp decline in vaccination rates over the past several years has been attributed to increasing amounts of vaccine hesitancy [9,10].

Due to various factors, vaccination rates among Hispanics rank lower than their White counterparts nationwide for many vaccines [11,12]. For example, as rates of cervical cancer in the US have decreased since the advent of the Human Papillomavirus (HPV) vaccine, Hispanic women are diagnosed with cervical cancer an average of 40% more often than non-Hispanic Whites, and they are 26% more likely to die from the disease [13]. Some studies have attributed this deficit to decreased insurance rates and a lack of consistent healthcare provider visits [14]. Others have cited an increased lack of medical knowledge among Hispanics, the COVID-19 pandemic, concerns about the safety of vaccines, and less connection to American culture [14–17].

The reasons behind vaccine hesitancy among Hispanics in the United States remain difficult to establish because Hispanic Immigrant communities are extremely diverse. Although it has been shown that vaccine uptake among Hispanics falls behind those of Whites nationwide, there is little evidence regarding reasons behind hesitancy among Hispanic Immigrants, specifically in relation to religious and folk influences [14,16,17]. To our knowledge, a study has yet to examine how these specific cultural factors influence vaccine hesitancy among Hispanics. Studying religious factors is especially important, as religious affiliation among the Hispanic community varies and many people cite religious exceptions to avoid vaccinating themselves or their children [18]. Additionally, previous studies found that culturally focused educational interventions are effective at influencing vaccine attitudes; understanding cultural influences would assist in the creation of culturally-specific vaccine interventions [19,20]. To better understand how cultural factors influence parental intent to vaccinate among Hispanic Immigrant communities across the nation, we surveyed parents directly.

## Materials and methods

### Survey description

Respondents completed a survey consisting of 116 questions. The survey measured vaccine attitudes, HPV knowledge, English language use, language barriers, trust in medicine/institutions, financial barriers, and demographics. Some questions were contingent on answers to previous questions; for example, only female participants were asked if they received regular pap smears. Questions were arranged in thirteen sections. The first section addressed demographic questions. The second section asked about the participant’s comfort speaking English and had a short English language evaluation. The third section requested information about the respondent’s access to healthcare in their preferred language. The fourth section assessed their knowledge of HPV. The fifth section asked questions regarding acculturation and assimilation to American culture. The sixth section determined financial and logistic barriers to vaccination. The seventh and eighth sections covered religious and social views, including a previously validated religiosity metric [19]. The ninth section measured the participants’ trust in institutions. The tenth and eleventh sections evaluated the respondent’s use of and attitudes toward traditional medicine. The twelfth section measured general vaccine attitudes (GVA). The last section asked questions about their HPV vaccination status. The entire survey is included in S1 Table.

The survey was administered both in English (231 responses) and Spanish (626 responses). Inclusion criteria included self-identification as Hispanic or Latino, identification as a country other than the United States as place of birth and having children.

SEM modeling requires at least 20 samples per variable [21]. Our models had 10 variables, so we needed at least 200 responses. We actually obtained and used 453 responses. 404 responses were discarded due to low data quality or because the participants did not fit the inclusion criteria (most of the excluded responses were not parents). 453 responses were used in the analysis. The survey was distributed in the United States through the Qualtrics platform (Provo, UT, USA).

### Human subjects

The study was approved by the Brigham Young University institutional review board (IRB2023-120). All subjects provided informed consent before beginning the study. The recruitment period was from May 31 2023 to June 11 2023.

### Factor analysis and structural equation modeling

In order to validate the latent variables in the survey, we used confirmatory factor analysis (CFA). Exploratory factor analysis (EFA) was used when a predefined structure was unclear. For each latent variable, CFA was performed with a request for modification indices and standardized coefficients. Survey items with low standardized p-values were removed until fit statistics (root mean square error approximation (RMSE), comparative fit index (CFI), Tucker–Lewis index (TLI) and standardized root mean square residual (SRMR)) were found to be in an acceptable range. All latent variables were represented by at least three items. To build a model and test the relationships between latent variables, we used structural equation modeling (SEM). Two SEM models were run with sex, income, and education as covariates. CFA and SEM were done using Mplus software, ver 8 (Munthen and Munthen, 1998–2001, Los Angeles, CA, USA).

## Results

### Demographics of survey respondents

**Fig 1:**
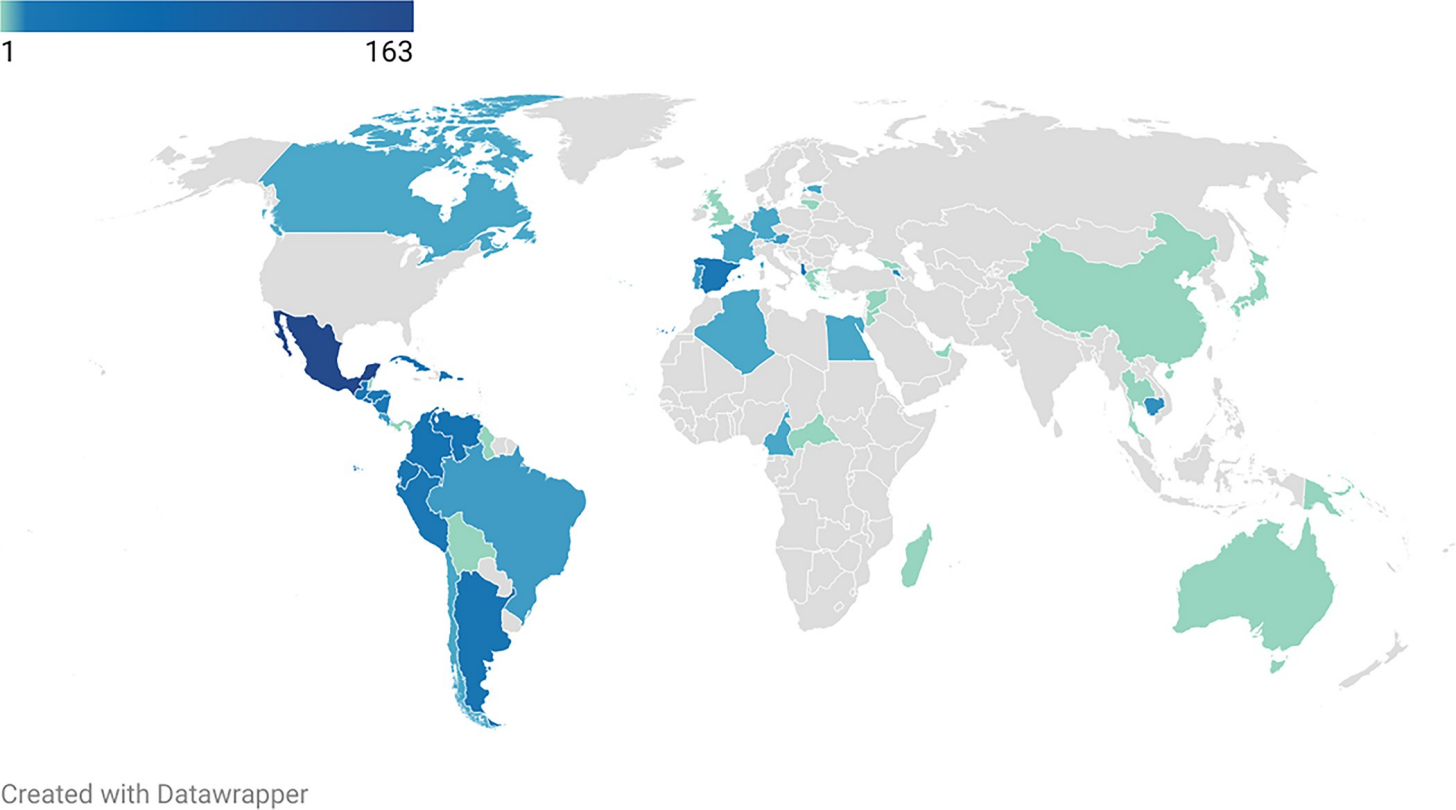
Map of participant birthplaces. This choropleth map shows frequencies of the birthplaces of the respondents. The most common countries of birth were Mexico (163 respondents), Puerto Rico (46 respondents), Cuba (31 respondents), Venezuela (25 respondents), and Colombia (22 respondents). 52 other countries were also represented. Map was generated using the Datawrapper tool, which uses OpenStreetMap maps as source files. OpenStreetMaps are open data https://www.openstreetmap.org/copyright.

Prior to formal analysis of survey data, we summarized the demographic characteristics of our sample (Table 1). Of the 453 survey responses in our data set, approximately three-fifths were female (61.5%) and two-fifths were male (37.9%). One respondent identified as nonbinary/other and one respondent preferred not to answer. All respondents were of Hispanic, Latino, or Spanish origin with the largest plurality specifically indicating they were of Mexican, Mexican American, or Chicano origin (45.2%). Survey responses indicated the data set was predominantly born in Mexico (34.4%), Puerto Rico (10.4%), Cuba (6.8%), Venezuela (5.7%), El Salvador (3.5%), Argentina (3.5%), or Dominican Republic (3.3%) (see **Fig 1**).

**Table 1:** Demographics. As a criterion for inclusion, all participants responded “yes” to a question which asked, “Are you of Hispanic, Latino, or Spanish Origin?” However, later in the survey, the participants were asked to specify their race/ethnicity. In this section, 14.5% did not specify that they identified as Hispanic. This could be due to confusion with the “choose all that apply” option.

Column1	Count	Percentage
Sex*		
** Male**	**172**	**38.1**
** Female**	**279**	**61.7**
** Non Binary/Other**	**1**	**0.22**
**Race (select all that apply)**		
** Hispanic/Latino**	**383**	**84.5**
** White**	**98**	**21.6**
** Black**	**40**	**8.8**
** Native American or Alaskan Native**	**17**	**3.8**
** Asian**	**15**	**3.3**
** Other**	**3**	**0.7**
**Age**		
** 18–25**	**90**	**19.9**
** 26–35**	**198**	**43.7**
** 36–45**	**139**	**30.7**
** 46+**	**26**	**5.7**
Income Level*		
** Less than $5,000**	**40**	**8.8**
** $5,000-$15,000**	**34**	**7.5**
** $16,000-$25,000**	**39**	**8.6**
** $26,000-$35,000**	**65**	**14.4**
** $36,000-$45,000**	**46**	**10.2**
** $45,000-$60,000**	**59**	**13.1**
** $61,000-$75,000**	**47**	**10.4**
** $76,000-$100,000**	**78**	**17.3**
** Over $100,000**	**44**	**9.7**
Marital Status*		
** Single**	**88**	**19.5**
** Partnered**	**96**	**21.2**
** Married**	**232**	**51.3**
** Divorced**	**26**	**5.8**
** Widow/Widower**	**8**	**1.8**
**Education**		
** Have not finished high school**	**22**	**4.9**
** Finished high school**	**85**	**18.8**
** Some college or vocational certificate**	**79**	**17.4**
** Associate’s degree**	**69**	**15.2**
** Bachelor’s degree**	**138**	**30.5**
** Advanced degree (masters, MD, DDS, PhD, etc.)**	**59**	**13.0**
**Number of Children**		
** 1**	**140**	**30.9**
** 2**	**198**	**43.7**
** 3**	**80**	**17.7**
** 4**	**20**	**4.4**
** 5+**	**15**	**3.3**

*responses do not add up to 453; not all participants responded to every question.

**Fig 2:**
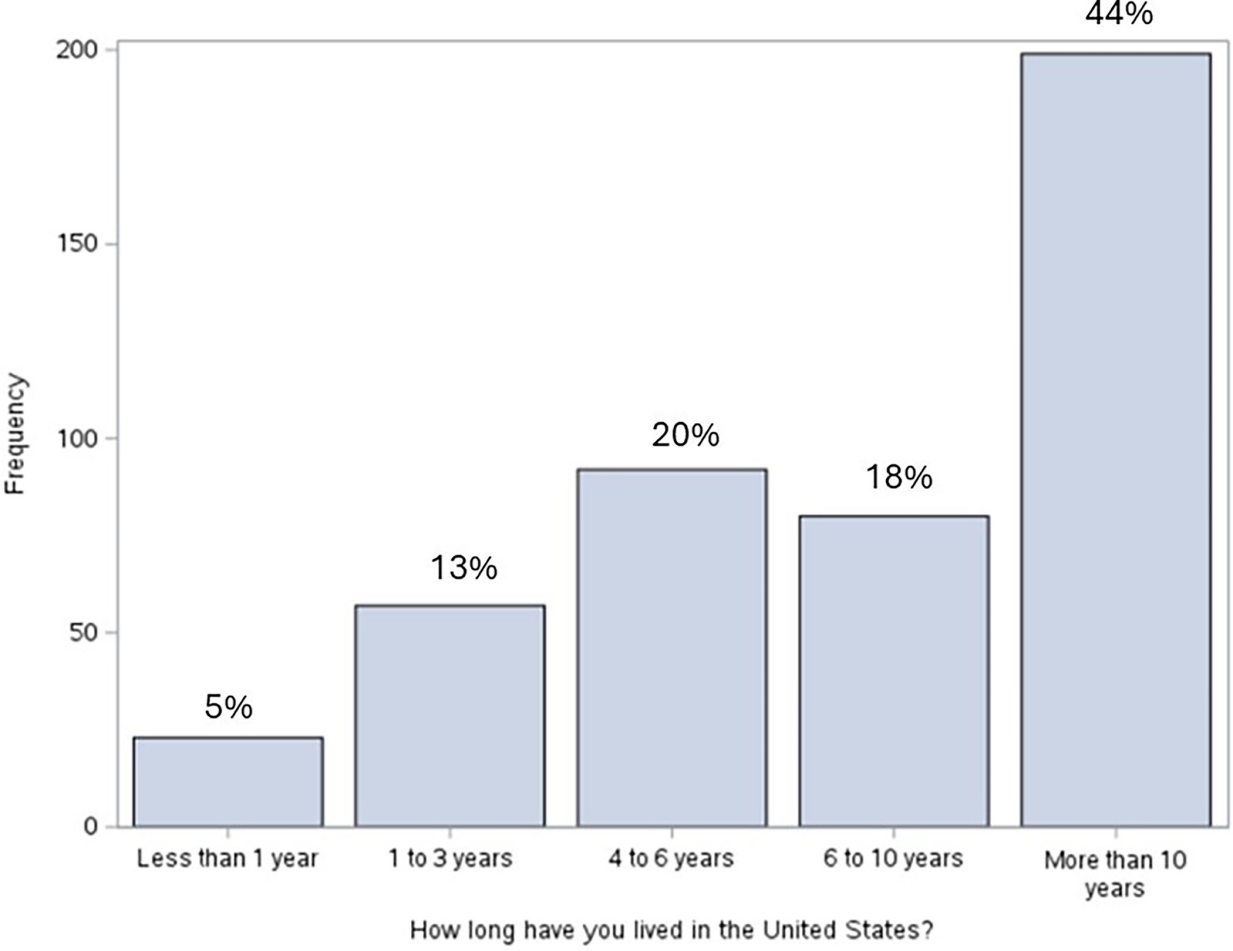
Time in the United States. Frequency of responses to the question, “How long have you lived in the United States?” The most common response was “More than 10 years” (44%). 18% of respondents said that they have lived in the US for 6 to 10 years, 20% have lived in the US for 4 to 6 years, 13% for 1 to 3 years, and 5 percent have only lived in the US for less than 1 year.

The majority of respondents were between ages 26–35 (43.7%) and ages 36–45 (30.7%). Approximately three-fourths of participants were either married (51.3%) or partnered (21.2%), and one-fourth were either single (19.5%), divorced (5.8%), or widowed (2.2%). Furthermore, the majority of the sample were young parents, which is likely related to the greatest number of respondents reporting having either 2 children (43.7%) or 1 child (30.9%). This is lower than the average number of children in Hispanic families, which is consistent with a sample predominantly containing young parents. Over half of participants indicated the majority of their family lives in the US (57.2%) while approximately two-fifths responded most of their family lives in their home country (37.8%). There was a wide variety of education levels in the study participants. While all of our sample were born in a foreign country, many of the respondents have lived in the United States for a long time (see **Fig 2**). A majority of our participants (62%) have lived in the United States for more than 6 years. 44% have lived in the US for more than 10 years. Only 5% have lived in the US for less than 1 year. Our participants are more recent immigrants than the population of immigrants in the US; 77% of Hispanic immigrants in the US have been in the country for 10 years or longer [22].

### Factor analysis

Confirmatory factor analysis (CFA) models were run for each latent variable in the hypothetical models for Structural Equation Modeling (SEM). Models were constructed to analyze cultural influences such as religious influence on vaccine attitudes (Model 1) and folk influence on vaccine attitudes (Model 2). All items fit the latent variables well in Model 1 (Table 2). In Model 2, exploratory factor analysis (EFA) indicated that the folklore items consisted of three factors that we labeled *Trust in Folk Medicine*, *Trust in Folk Practitioners*, and *Use of Home Remedies*. One item, “I trust in folk (traditional medicine, curanderismo)”, did not fit any factor and was removed. EFA on *Trust in Institutions* indicated two factors, but one factor only had two items, “My medical provider cares about my health” and “My medical provider only cares about making money”, thus the items were removed. Additionally, CFA on the remaining items indicated a low factor loading for the item, “The companies that make vaccines want to make money at the expense of my heath”, and it was removed. The remaining items comprised the latent variable *Trust in Institutions* (Table 2).

**Table 2:** Fit statistics for item general vaccine attitudes. Please see S1 Table for the full CFA table.

Model (Latent Variables)	RMSEA	CFI	TLI	SRMR
Model 1	0.052	0.928	0.915	0.055
Model 2	0.053	0.923	0.912	0.051

### Structural equation modeling

**Fig 3:**
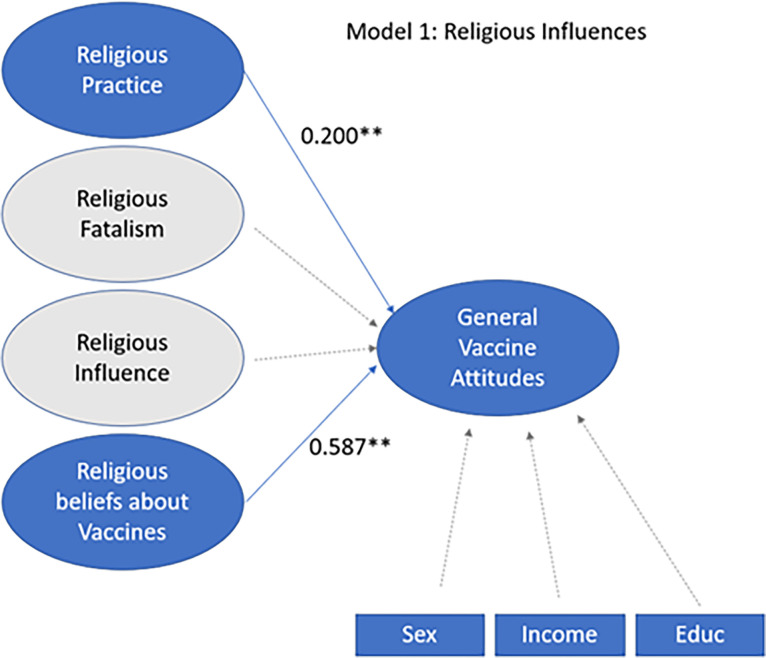
Religious Influences Model. Latent variables *Religious Practice*, *Religious Fatalism*, *Religious Influence*, *Religious beliefs about Vaccines*, and *General Vaccine Attitudes* were combined in a structural model. Higher religious practice leads to more positive attitudes towards vaccination. Likewise, having religious beliefs that support vaccination is positively correlated with positive vaccine attitudes. Religious fatalism and religious influence on vaccination are not correlated with vaccine attitudes. Values shown are standardized beta coefficients. Values with one asterisk are significant at p< 0.05. Values with two asterisks are highly significant, at p≤0.01.

SEM for Model 1 indicates Religious beliefs about Vaccines (+0.587) was the strongest predictor of greater GVA. Additionally, greater Religious Practice predicts greater GVA (+0.200). There was no significant relationship shown between Religious Fatalism and GVA as well as between Religious influence and GVA. There was no significant relationship between GVA and Sex, Education, or Income (**Fig 3**).

**Fig 4:**
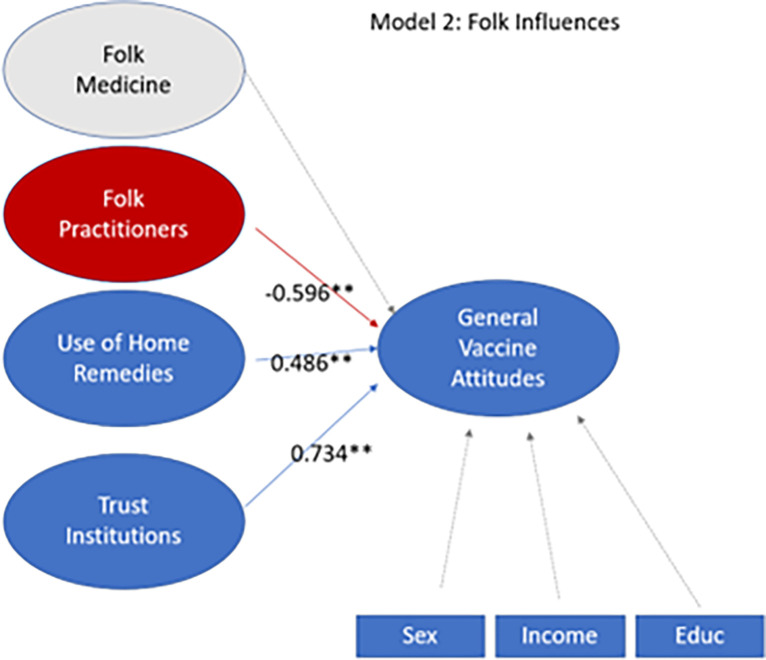
Folk influences model. Latent variables *Folk Medicine*, *Folk Practitioners*, *Use of Home Remedies*, *Trust in Institutions*, and *GVA* were combined into a structural model. The belief in various Hispanic medicine folklore was not significantly related to vaccine attitudes. Participants having more trust in a practitioner of folk medicine than a medical doctor had a significant inverse relationship with positive vaccine attitudes. However, the use of home remedies was positively correlated with GVA. High trust in institutions led to high vaccine attitudes. Income, education, and sex did not impact GVA. Values shown are standardized beta coefficients. Values with one asterisk are significant at p< 0.05. Values with two asterisks are highly significant, at p≤0.01.

SEM on Model 2 (see **Fig 4**) reflects folk medicine influences on vaccine attitudes. Trust in Institutions (+0.734) was the strongest predictor of greater GVA. GVA was also related to Use of Home Remedies (+0.486). However, Model 2 supports an inverse relationship between GVA and trust in practitioners of folk medicine (-0.596). There was no significant relationship between the latent variable *Folk Medicine* and GVA. Additionally, there was no significant relationship between GVA and Sex, Education, or Income.

**Fig 5:**
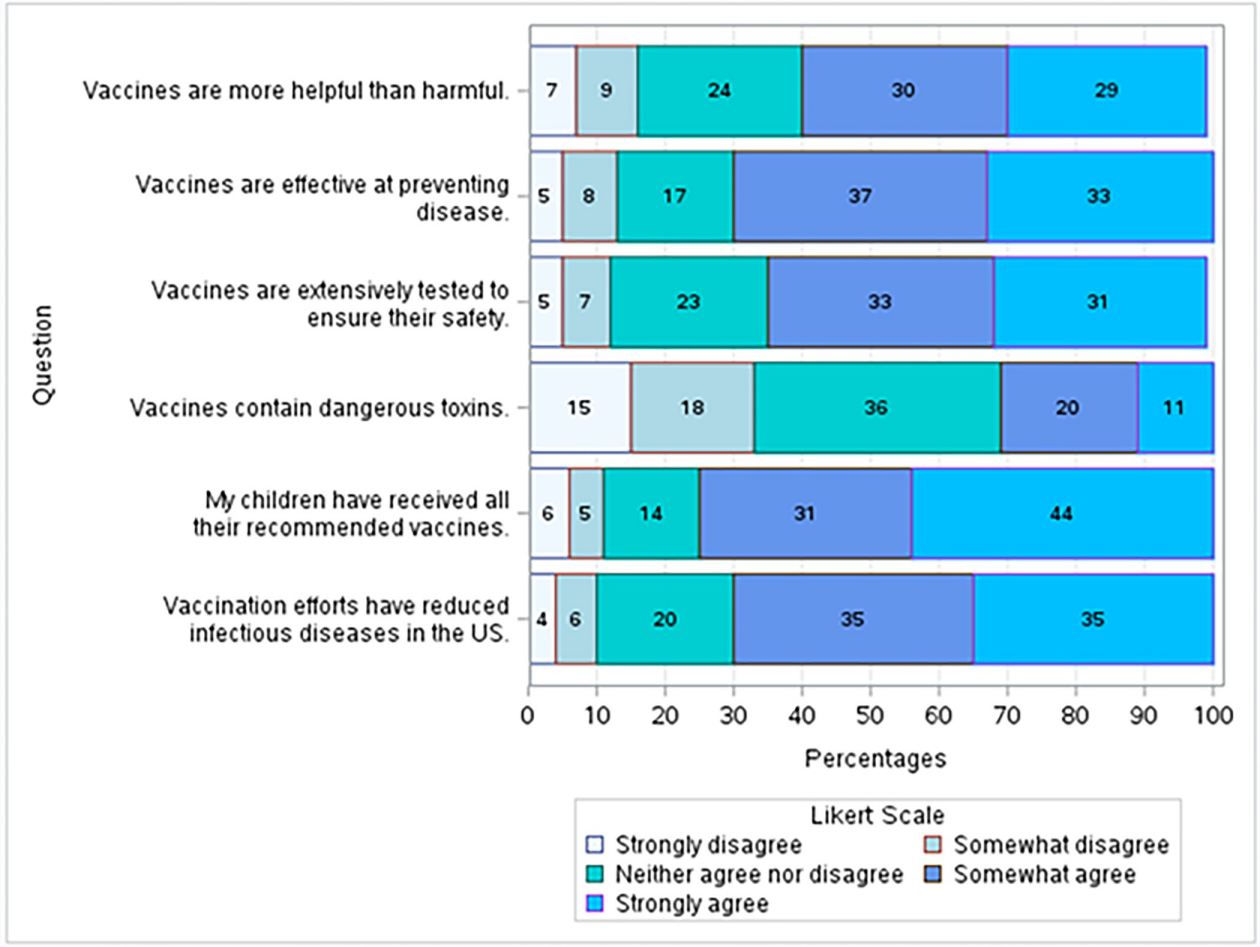
Vaccine attitudes. Six survey items were used to measure GVA. Respondents tended to have pro-vaccination attitudes, with about 30% of respondents responding “strongly agree” to the statements “Vaccines are more helpful than harmful,” “Vaccines are effective at preventing disease,” “Vaccines are extensively tested to ensure their safety,” and “Vaccination efforts have reduced infectious diseases in the US.” Only a small percentage (4–7%) responded “strongly disagree” to those four statements. The question “Vaccines contain dangerous toxins” had a higher percentage of participants respond “strongly disagree,” and more than a third (36%) responded “Neither agree nor disagree.” A plurality (44%) responded “strongly agree” to the question “My children have received all of their recommended vaccines.” Only 11% responded “strongly disagree” or “somewhat disagree”.

Six survey questions were used to develop GVA. The proportions of answers to each question are shown in **Fig 5.** Three of the questions tested the participant’s perception of the helpfulness of vaccines (“Vaccines are more helpful than harmful,” “Vaccines are effective at preventing disease,” and “Vaccination efforts have reduced infectious diseases in the US.”) More than 50% of participants somewhat agreed or strongly agreed with each of those statements; between 16%, 13%, and 10% responded “Somewhat disagree” or “Strongly disagree” to each of the questions, respectively. Two questions evaluated the respondent’s perception of the safety of vaccines (“Vaccines are extensively tested to ensure their safety,” and “Vaccines contain dangerous toxins”). 64% of participants somewhat agreed or strongly agreed that vaccines are tested to ensure their safety, and only 12% somewhat disagreed or strongly disagreed. 33% responded “Strongly disagree” or “Somewhat disagree” that vaccines contain dangerous toxins. 31% somewhat agreed or strongly agreed. It is important to note that “Vaccines contain dangerous toxins” was reverse-coded to check for acquiesce bias.

**Fig 6:**
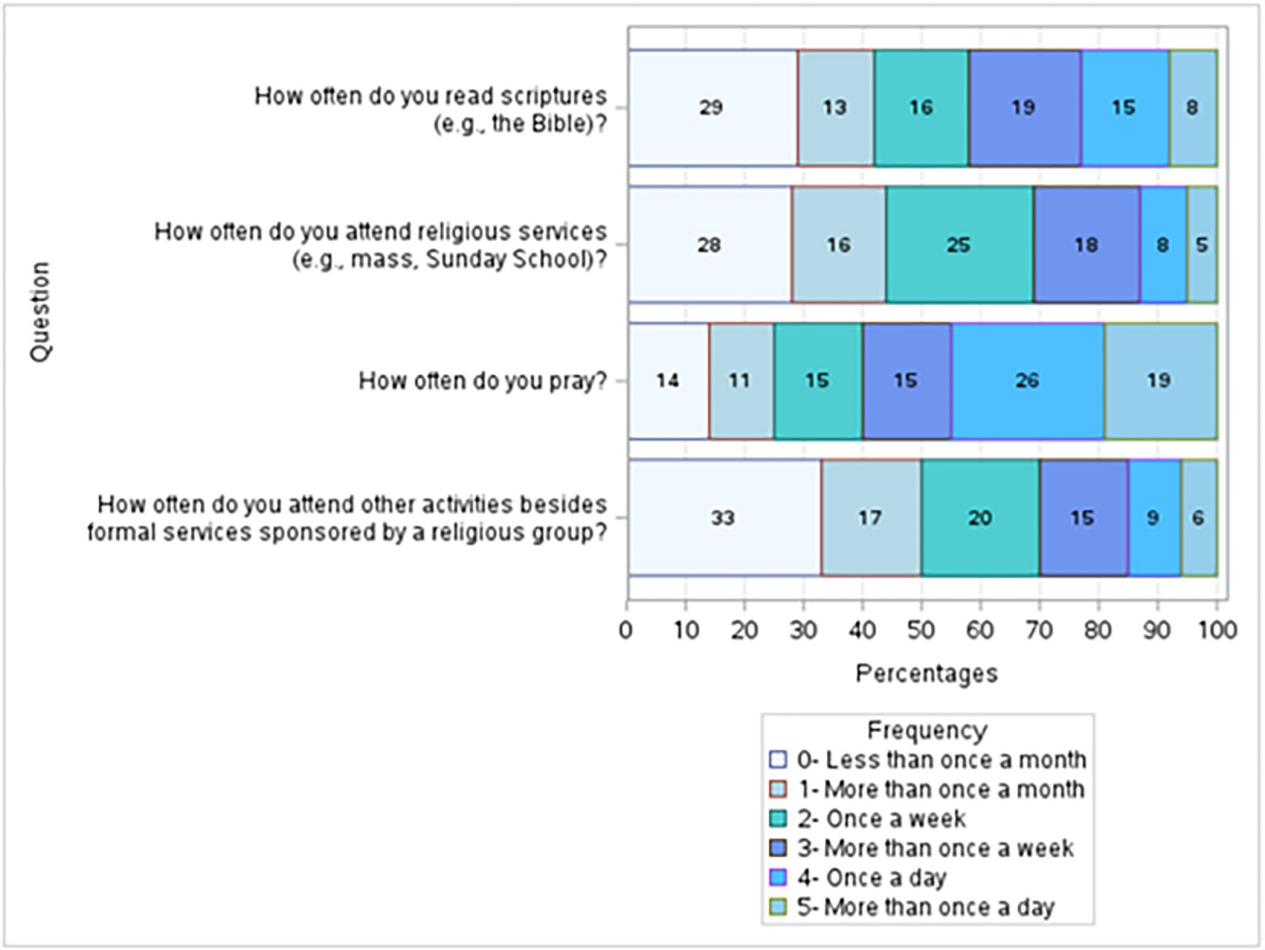
Religious Activity. Respondents were active in organized religion, with 56% and 50% responding that they attend religious services or other religious activities sponsored by a religious group, respectively. 42% of respondents reported reading scriptures more than once a week, once a day, or more than once a day. The religious activity most engaged in by our sample was prayer. 45% of the respondents reported praying once a day or more than once a day; an additional 30% pray at least once a week.

Six survey questions were used to develop the variable *Religious Practice*. Two of the questions, “How often do you attend religious services?” and “How often do you attend other activities besides formal services sponsored by a religious group?” aimed to evaluate the respondent’s participation in organized religion. Our sample is very active in organized religion. Only 28% and 33%, respectively, of participants responded that they attend religious services or activities sponsored by a religious group less than once a month; 56% and 50%, respectively responded that they attend religious services or activities sponsored by a religious group once a week or more. The other two questions, “How often do you read the scriptures?” and “How often do you pray?” measured the participants’ private worship habits. While 29% of respondents reported reading religious books less than once a month, 58% read once a week or more. Even more participants pray: only 14% reported praying less than once a month, and 75% pray once a week or more. 19% pray more than once a day (**Fig 6**).

**Fig 7:**
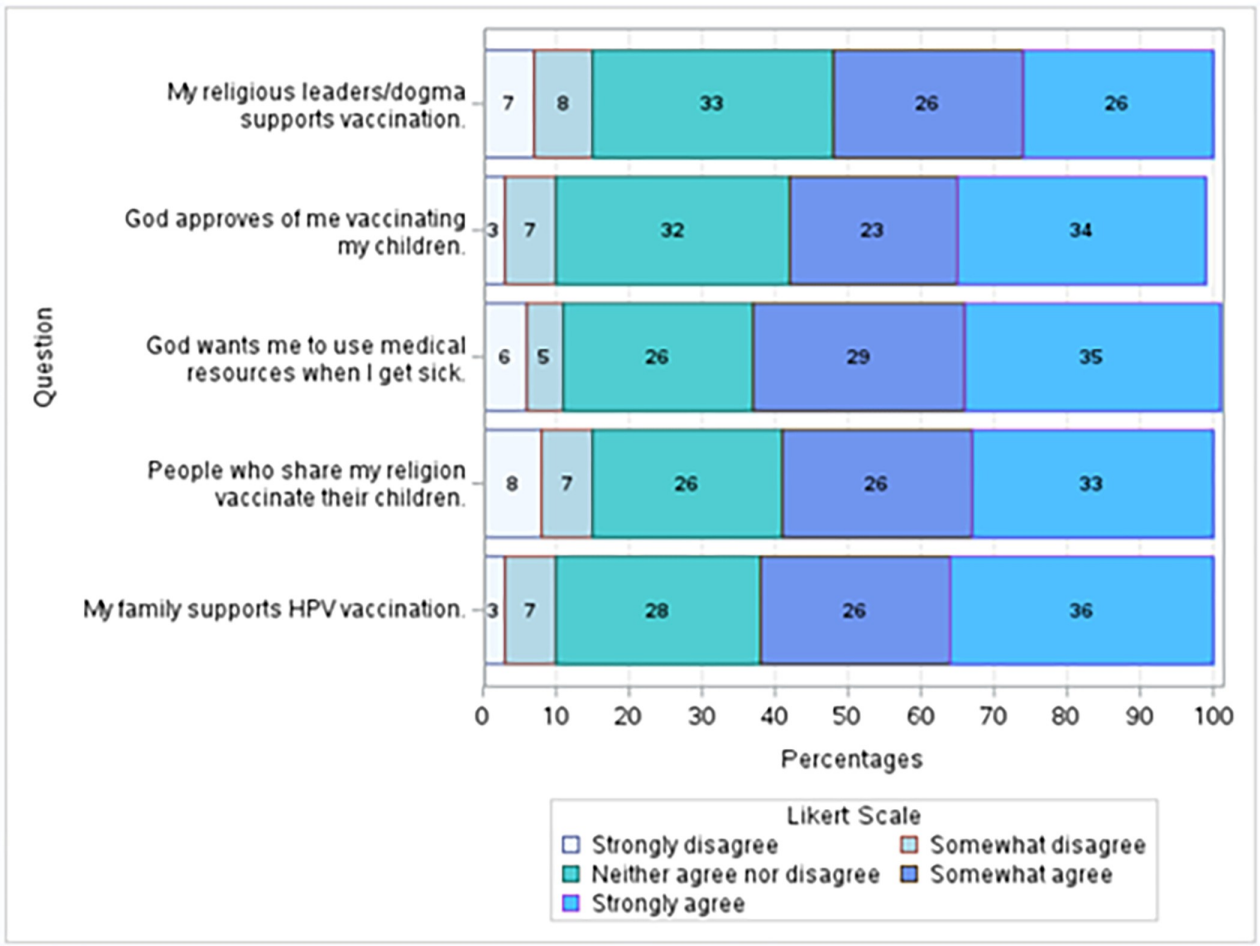
Social and religious views towards vaccination. 52% of respondents responded “Strongly agree” or “Somewhat agree” to whether their religious leaders support vaccination. 59% of participants either strongly agreed or agreed that people who share their religion vaccinate their children. To understand the participants’ theological view of vaccination and medical care, they were to rate their level of agreeance to the questions, “God approves of me vaccinating my children,” and “God wants me to use medical resources when I get sick.” Again, most participants either agreed or strongly agreed with these statements (57% and 64%, respectively). Lastly, the participants were asked if their family supports HPV vaccination. 62% responded “Strongly agree” or “Somewhat agree,” and only 3% responded “Strongly disagree”.

Five questions were used to form the variable “Religious beliefs about vaccines.” Our sample, for the most part, tends to agree that their religious leaders, the people who share their religion, and their families support vaccination. When asked if their religious leaders/dogma support vaccination, 52% agreed or strongly agreed; only 15% disagreed or strongly disagreed. Even more of the participants reported that they think God approves of them vaccinating their children: 57% of participants responded “Strongly agree” or “Somewhat agree,” and only 10% responded “Strongly disagree” or “Somewhat disagree.” Similarly, most participants said that God wants them to use medical resources when they are sick. The religious acceptance of vaccines extends to social acceptance. 59% of individuals reported that they “Strongly agree” or “Somewhat agree” with the statement “People who share my religion vaccinate their children.” When asked if their family supports HPV vaccination, 62% agreed or strongly agreed **(Fig 7**).

**Fig 8:**
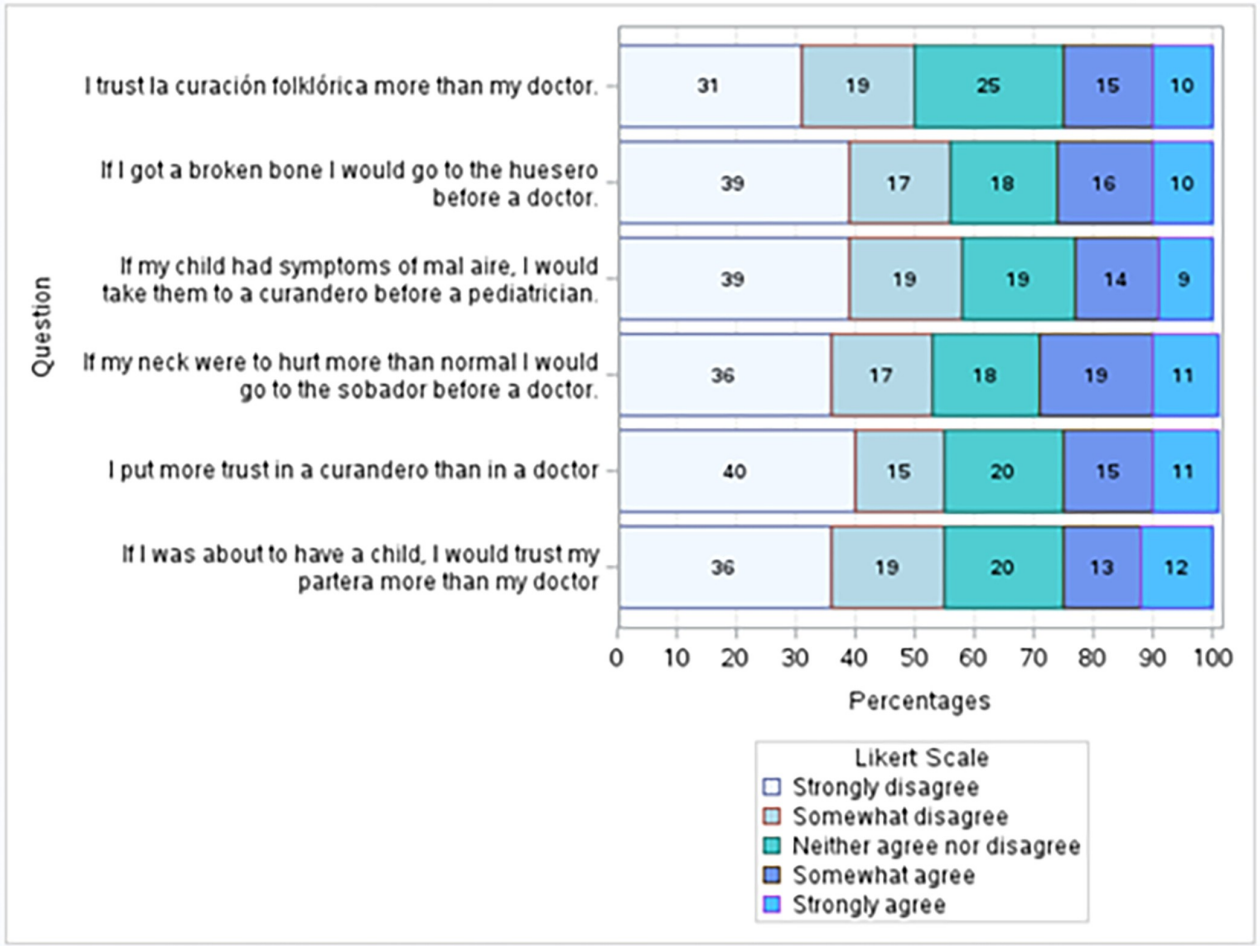
Trust in types of practitioners. Participants were asked six questions to evaluate their level of trust towards medical doctors as opposed to folk practitioners. In response to the first question, “I trust curación folklórica [folk medicine] more than my doctor,” 50% of participants disagreed or strongly disagreed. However, 25% agreed or strongly agreed. Similarly, when asked if the participants “put more trust in a curandero [healer] than in a doctor,” 55% disagreed or strongly disagreed; 26% agreed or strongly agreed. The rest of the questions listed symptoms (broken bones, mal aire, hurt neck, imminent childbirth), and were asked their level of agreeance on a statement that they would rather go to a specific folk medicine doctor, or a medicine doctor. While most (58%-53%) reported that they disagreed or strongly disagreed with the statements, between 23% 30% agreed or strongly agreed.

Six questions were used to develop the variable *Folk Practitioners*. The proportions of answers to each question are shown in **Fig 8.** When asked if they put more trust in folk medicine or folk practitioners than their doctors, 55% and 50%, respectively, responded “Strongly disagree” or “Somewhat disagree.” About a quarter (25% and 26%) of respondents either agreed or strongly agreed with those two statements. The participants were also asked four questions about where they would go for medical help: “If I got a broken bone I would go to the huesero [folk practitioner specializing in bone fractures] before a doctor;” “If my child had symptoms of mal aire [bad aire], I would take them to a curandero [healer] before a pediatrician;” “If my neck were to hurt more than normal I would go to the sobador [masseur] before the doctor;” and “If I were about to have a child, I would trust my partera [midwife] more than my doctor.” Most participants responded “Strongly disagree” or “Somewhat disagree” to these statements. 30% to 25% agreed or strongly agreed.

## Discussion

### Religious practice and beliefs increases vaccine attitudes

The model in **Fig 2** suggests that high religious practice and positive religious belief toward vaccination are predictive of high general vaccine attitudes. Our study proposes that Hispanic immigrants with more religiosity are more likely to have positive attitudes toward vaccination. Additionally, those who believe that their religion or people who share their religion support vaccination are also likely to support vaccination. Past studies have shown that the relationship between religion/faith and vaccination is complex, and some studies have found a negative or no association between religion and vaccine attitudes among Hispanics [23–25]. However, in a study of religious individuals in the United States, Redd and colleagues found a complicated, yet positive, relationship between religiosity and pro-HPV vaccine attitudes [26]. Our findings may indicate that if a respondent believes their religion supports vaccination (which was the majority of our respondents), the highly religious are more likely to be vaccinated. This suggests that including religion in positive discussion of vaccinations may be a way to overcome a barrier towards vaccination. This association between faith and medicine highlights the importance for health officials to partner with faith leaders in Hispanic communities to decrease vaccination disparities.

### High trust in folk practitioners decreases GVA

Trusting folk practitioners (curanderos) more than medical practitioners is correlated with significantly lower attitudes towards vaccination, as shown in **Fig 3.** This follows, as folk practitioners may be financially motivated to direct their patients away from traditional medicine. Similar findings have been explored in regard to Complementary Alternative Medicine (CAM) practitioners. Dr. William Bleser, PhD, found that children who visited with CAM practitioners were less likely to receive the influenza vaccine [27]. CAM practitioners have been shown to be less likely to recommend vaccination than non-CAM pediatricians [28]. Less research has been conducted regarding the vaccine attitudes of curanderos. More research needs to be performed to understand this relationship.

### The use of home remedies increases GVA

Model 2 (**Fig 3**) shows that the use of home remedies is indicative of positive vaccine attitudes. Fowler, et al found that 83% of Hispanic parents in their study used cultural remedies with their children; foreign-born parents were more likely than US-born parents to participate in home remedies. A common practice among the Hispanic immigrant community is “medical pluralism,” meaning that health beliefs and practices are a synthesis of cultural, traditional, religious, and scientific sources [29]. Our study provides an example of medical pluralism, in that the Hispanic immigrant parents sampled were likely to accept both home remedies and vaccination as acceptable and non-contradictory.

### Trust in institutions is strongly associated with high GVA

In model 2 (**Fig 3**), trust in institutions is the greatest indicator of GVA. This confirms previous findings that show trust in the scientific community and other institutions of authority to be the greatest or one of the greatest predictors of vaccination [30–32]. As stated by Heidi Larson, et al, trust and attitudes towards vaccines "exist within the additional context of deeper, underlying trust in society at large." [33]. For Hispanic immigrants, whose lives are impacted by ever-changing legislation, including regarding vaccine requirements for immigrants, the effects of trust towards institutions on vaccine attitudes are compounded. Additionally, children’s’ vaccination requirements are determined by the individual states and school districts; an immigrant’s trust in both the states and school district in making choices that will be beneficial for their child may determine their attitudes towards receiving the vaccines.

Trust may be more difficult to foster among undocumented immigrants, who may distrust and be fearful of the government. All institutions, though especially smaller institutions, such as schools and medical providers, must work to create relationships of trust with Hispanic immigrants and other vulnerable populations.

### Limitations

Due to the online distribution of our survey, individuals without internet access or who do not respond to surveys in this manner would be excluded. The sample size was also relatively small and may not represent the entire population. Mexican origin represented 34% of responses, while Mexican immigrants make up 53% of immigrants in the United States [22]. Several Spanish-speaking countries were not represented at all in our sample, or in very small numbers. As the culture of every Spanish-speaking country can be starkly different, we were only able to investigate the cultures of the countries from which we received responses. Though every participant of our survey responded that they identified as Hispanic or Latino, 57 countries were reported, many of them not of Hispanic origin. While there is a diaspora of Spanish-speaking immigrants from all over the world, meaning a person of Hispanic descent could be born in a country that is not Spanish-speaking, our results could be due to a small number of participants misrepresenting their ethnicity or birthplace. Lastly, this project originally attempted to measure attitudes towards the HPV vaccine, but several of the variables measuring HPV attitudes did not fit well in the models and had to be discarded, despite good fit in other studies [17,26]. Therefore, our findings are not HPV-specific. We are currently carrying out focus groups to identify HPV-specific obstacles for future investigation.

## Conclusions

Religious practice and religious beliefs about vaccines are positively correlated with high vaccine attitudes. While having more trust in folk practitioners than medical doctors decreases vaccine attitudes, the use of home remedies is correlated with positive attitudes toward vaccination. High trust in institutions is predictive of high vaccine attitudes. Medical training, including training of personnel at all levels, such as vaccine clinic workers, could focus on cultural competency and the training of providers within these cultures to improve trust and fluency in communication.

The finding that using home remedies was correlated with positive attitudes towards vaccination should encourage medical professionals to become more familiar with and knowledgeable about traditional Hispanic remedies. As medical professionals become more culturally competent with the cultures of their patients, the relationship of trust will grow, therefore increasing vaccine attitudes.

Future work in this area could include qualitative studies to delve deeper into the relationship between the cultural factors identified as significant and vaccine attitudes, as well as the development and testing of culturally competent educational materials. Other potential work could include working with folk medicine practitioners and religious leaders to foster trust in vaccines and public health programs.

## Supporting information

S1 Table Confirmatory factor and reliability analysis for survey latent variables all factor loadings are significant at p < .001. (DOCX)

S1 Data Complete data file. (XLSX)
