# Supplementary material for: Effects of religious and cultural beliefs on vaccine attitudes in a Hispanic immigrant population in the United States
Source: PLOS Glob Public Health. 2024 Aug 6;4(8):e0003519. doi: 10.1371/journal.pgph.0003519 (PMC11302901; doi:10.1371/journal.pgph.0003519)
Supplement: S1 Table — (DOCX) [file pgph.0003519.s001.docx]

# Supplementary Materials

**S1 Table**. Confirmatory Factor and Reliability Analysis for Survey Latent Variables All factor loadings are significant at p<.001.

| **Constructs** | **Factor Loading** |
| --- | --- |
| *English Proficiency** & *Acculturation*  Fit: RMSEA = 0.066, CFI = 0.962, TLI = 0.945, SRMR = 0.068  Correlated errors: Item 1 with Item 2  Scale | |
| *English Proficiency* | |
| ¿Qué tan cómodo se siente hablando inglés en la escuela? | 0.707 |
| ¿Qué tan cómodo se siente hablando inglés en el trabajo? | 0.715 |
| Elija la opción que mejor se adapte a su nivel de comodidad. - ¿Qué tan cómodo se siente hablando inglés en casa? | 0.761 |
| Elija la opción que mejor se adapte a su nivel de comodidad. - ¿Qué tan cómodo se siente hablando inglés en público? | 0.899 |
| Elija la opción que mejor se adapte a su nivel de comodidad. - ¿Qué tan cómodo se siente al entender inglés? | 0.761 |
| *Acculturation* | |
| ¿Cuánto tiempo ha vivido en los Estados Unidos? | 0.359 |
| Sírvase indicar la mejor respuesta. - Me veo a mí mismo como parte de la cultura estadounidense. | 0.896 |
| Sírvase indicar la mejor respuesta. - Me veo a mí mismo como un estadounidense. | 0.636 |
| Sírvase indicar la mejor respuesta. - Continúo con muchas de las tradiciones o formas de mi país de origen. | -0.332 |
| *Access to Healthcare in Preferred Language*  Fit: RMSEA = 0.056, CFI = 0.953, TLI = 0.940, SRMR = 0.047 | |
| *Receive Healthcare in Spanish* | |
| Sírvase indicar la mejor respuesta. - Me siento más cómodo cuando mi médico habla español. | 0.688 |
| Sírvase indicar la mejor respuesta. - Siento que la atención que recibo no es tan eficaz cuando mi médico sólo habla inglés. | 0.642 |
| Sírvase indicar la mejor respuesta. - Si mi médico hablara español, me resultaría más fácil confiar en él. | 0.762 |
| Sírvase indicar la mejor respuesta. - Me resulta confuso el sistema de atención médica en los Estados Unidos. | -0.514 |
| *Trust in Language-Specific Healthcare* | |
| Sírvase indicar la mejor respuesta. - Tengo una buena relación con mi médico. | 0.754 |
| Sírvase indicar la mejor respuesta. - Confío en lo que mi médico me dice que haga. | 0.785 |
| Sírvase indicar la mejor respuesta. - Siento que tengo acceso a suficiente información cuando tengo que tomar decisiones de salud. | 0.680 |
| Sírvase indicar la mejor respuesta. - Los consultorios médicos y los hospitales cuentan con suficientes recursos de traducción. | 0.612 |
| Sírvase indicar la mejor respuesta. - Las fuentes que utilizo actualmente para la información de salud ayudan a responder mis preguntas. | 0.727 |
| Sírvase indicar la mejor respuesta. - Confío más en la información que me dan mis médicos que en los recursos en línea que utilizo. | 0.710 |
| Sírvase indicar la mejor respuesta. - Confío en que mi médico comprende mis necesidades de atención médica. | 0.732 |
| *HPV Knowledge* & General Vaccine Attitudes*  Fit: RMSEA = 0.063, CFI = 0.959, TLI = 0.943, SRMR = 0.043 | |
| *HPV Knowledge* | |
| Sírvase indicar la mejor respuesta. - El VPH es una infección potencialmente mortal. | 0.744 |
| Sírvase indicar la mejor respuesta. - La infección por VPH puede causar un sufrimiento físico grave. | 0.820 |
| Sírvase indicar la mejor respuesta. - Solo una pequeña minoría de personas contraerá el VPH durante su vida. | -0.396 |
| Sírvase indicar la mejor respuesta. - El VPH causa una cantidad sustancial de cáncer en las mujeres y los hombres. | 0.560 |
| *General Vaccine Attitudes* | |
| Las vacunas son más útiles que dañinas. | 0.706 |
| Las vacunas son eficaces para prevenir enfermedades. | 0.872 |
| Las vacunas son ampliamente evaluadas para garantizar su seguridad. | 0.829 |
| Mis hijos han recibido todas sus vacunas recomendadas. | 0.698 |
| Los esfuerzos de vacunación han reducido las enfermedades infecciosas en los  Estados Unidos. | 0.729 |
| *Barriers to Vaccination*  Fit: RMSEA = 0.058, CFI = 0.973, TLI = 0.957, SRMR = 0.036 | |
| *Affordability Barriers* | |
| Tengo un seguro médico adecuado. | 0.672 |
| Sírvase indicar la mejor respuesta. - Mi seguro médico generalmente cubre la vacunación. | 0.713 |
| Sírvase indicar la mejor respuesta. - Mi seguro médico cubre la vacunación contra el VPH. | 0.813 |
| Sírvase indicar la mejor respuesta. - Recibir la vacuna contra el VPH sería asequible para mí. | 0.722 |
| *Location Barriers* | |
| Sírvase indicar la mejor respuesta. - Sé adónde ir para vacunarme. | 0.874 |
| Sírvase indicar la mejor respuesta. - Sé cómo encontrar mi departamento de salud local. | 0.783 |
| ¿Cuánto tiempo le llevaría llegar a un lugar que ofrezca vacunas? | -0.220** |
| *Religiosity*  Fit: RMSEA = 0.078, CFI = 0.933, TLI = 0.914, SRMR = 0.062 | |
| *Religious Practice* | |
| Por favor, indique con qué frecuencia realiza estas prácticas. - ¿Con qué frecuencia lee escrituras (p. ej., la Biblia, el Corán)? | 0.758 |
| Por favor, indique con qué frecuencia realiza estas prácticas. - ¿Con qué frecuencia asiste a servicios religiosos (p. ej., misa, la mezquita. la escuela dominical)? | 0.860 |
| Por favor, indique con qué frecuencia realiza estas prácticas. - ¿Con qué frecuencia ora? | 0.380 |
| Por favor, indique con qué frecuencia realiza estas prácticas. - ¿Con qué frecuencia asiste a otras actividades además de los servicios formales patrocinados por un grupo religioso? | 0.686 |
| *Religious Influence* | |
| Por favor, indique cuánta influencia tienen sus creencias religiosas en estas decisiones. - ¿Cuánta influencia tienen sus creencias religiosas en lo que decide vestir? | 0.776 |
| Por favor, indique cuánta influencia tienen sus creencias religiosas en estas decisiones. - ¿Cuánta influencia tienen sus creencias religiosas en lo que decide comer y beber? | 0.859 |
| Por favor, indique cuánta influencia tienen sus creencias religiosas en estas decisiones. - ¿Cuánta influencia tienen sus creencias religiosas en sus decisiones sobre su salud? | 0.874 |
| Por favor, indique cuánta influencia tienen sus creencias religiosas en estas decisiones. - ¿Cuánta influencia tienen sus creencias religiosas en las actividades sociales que realiza? | 0.824 |
| Por favor, indique cuánta influencia tienen sus creencias religiosas en estas decisiones. - ¿En qué medida sus creencias religiosas afectan las decisiones importantes que toma? | 0.801 |
| *Religious Fatalism* | |
| Sírvase indicar la mejor respuesta. - Cuando alguien se enferma es porque Dios le dio esa enfermedad. | 0.883 |
| Sírvase indicar la mejor respuesta. - Cuando alguien se enferma es porque Dios lo está castigando. | 0.902 |
| Sírvase indicar la mejor respuesta. - Dios quiere que confíe principalmente en la fe y la oración para recuperarme de las enfermedades. | 0.586 |
| *Religious Influence on Vaccines*  Fit: RMSEA = 0.042, CFI = 0.993, TLI = 0.985, SRMR = 0.019 | |
| Sírvase indicar la mejor respuesta. - Mis líderes religiosos/el dogma apoyan la vacunación. | 0.664 |
| Sírvase indicar la mejor respuesta. - Dios aprueba que vacune a mis hijos. | 0.807 |
| Sírvase indicar la mejor respuesta. - Dios quiere que use recursos médicos cuando me enferme. | -0.823 |
| Sírvase indicar la mejor respuesta. - Las personas que comparten mi religión vacunan a sus hijos. | 0.777 |
| Sírvase indicar la mejor respuesta. - Mi familia apoya la vacunación contra el VPH. | 0.685 |
| *Trust in Institutions*  Fit: RMSEA = 0.058, CFI = 0.983, TLI = 0.967, SRMR = 0.026 | |
| La escuela de mi hijo se preocupa por su salud. | 0.635 |
| El Gobierno de los Estados Unidos se preocupa por mi salud. | 0.712 |
| Cuando la Administración de Alimentos y Medicamentos (Food and Drug  Administration, FDA) aprueba una vacuna, puedo confiar en que es  segura. | 0.703 |
| Cuando el Gobierno de los Estados Unidos recomienda que reciba una  vacuna, me la pongo. | 0.752 |
| Las compañías que producen vacunas se preocupan por mi salud. | 0.759 |
| *Folklore*  Fit: RMSEA = 0.068, CFI = 0.937, TLI = 0.925, SRMR = 0.049 |  |
| *Trust in Folk Medicine* | |
| Creo que el bautismo puede prevenir el mal aire. | 0.960 |
| La limpia con huevo ha sido un tratamiento válido en mi vida. | 0.787 |
| He ido a un sobador varias veces en mi vida. | 0.715 |
| Alguien que conozco ha sufrido del mal de ojo. | 0.767 |
| El mal de ojo se puede curar usando una limpia con huevo. | 0.826 |
| Alguien que conozco ha sufrido de un susto. | 0.726 |
| En general, confío en la curación folklórica. | 0.772 |
| *Trust in Folk Practitioners* | |
| Confío más en la curación folklórica que en mi médico. | 0.801 |
| Si me fracturara un hueso, iría al huesero antes que al médico. | 0.819 |
| Si mi hijo tuviera síntomas de mal aire, lo llevaría a un curandero antes  que al pediatra. | 0.856 |
| Si me doliera el cuello más de lo normal, iría al sobador antes que al  médico. | 0.851 |
| Confío más en un curandero que en un médico. | 0.897 |
| Si estuviera a punto de tener un hijo, confiaría más en mi partera que en  mi médico. | 0.817 |
| *Home Remedies* | |
| Creo que los remedios caseros son formas válidas de tratamiento para las  enfermedades. | 0.677 |
| Mi familia tiene una tradición de remedios caseros. | 0.674 |
| Confío más en mis remedios caseros que en mi médico. | 0.833 |

* Ran together with another latent variable due to a perfect-fitting model when run alone

** Despite the low factor loading, we chose to retain the item to maintain the latent variable
